# In silico identification of novel biomarkers for key players in transition from normal colon tissue to adenomatous polyps

**DOI:** 10.1371/journal.pone.0267973

**Published:** 2022-04-29

**Authors:** Zerrin Isik, Asım Leblebici, Ezgi Demir Karaman, Caner Karaca, Hulya Ellidokuz, Altug Koc, Ender Berat Ellidokuz, Yasemin Basbinar

**Affiliations:** 1 Faculty of Engineering, Department of Computer Engineering, Dokuz Eylul University, Izmir, Turkey; 2 Department of Translational Oncology, Institute of Health Sciences, Dokuz Eylul University, Izmir, Turkey; 3 Department of Computer Engineering, Institute of Natural and Applied Sciences, Dokuz Eylul University, Izmir, Turkey; 4 Department of Preventive Oncology, Institute of Oncology, Dokuz Eylul University, Izmir, Turkey; 5 Gentan Genetic Medical Genetics Diagnosis Center, Izmir, Turkey; 6 Faculty of Medicine, Department of Gastroenterology, Dokuz Eylul University, Izmir, Turkey; 7 Department of Translational Oncology, Institute of Oncology, Dokuz Eylul University, Izmir, Turkey; University of Oklahoma Health Sciences Center, UNITED STATES

## Abstract

Adenomatous polyps of the colon are the most common neoplastic polyps. Although most of adenomatous polyps do not show malign transformation, majority of colorectal carcinomas originate from neoplastic polyps. Therefore, understanding of this transformation process would help in both preventive therapies and evaluation of malignancy risks. This study uncovers alterations in gene expressions as potential biomarkers that are revealed by integration of several network-based approaches. In silico analysis performed on a unified microarray cohort, which is covering 150 normal colon and adenomatous polyp samples. Significant gene modules were obtained by a weighted gene co-expression network analysis. Gene modules with similar profiles were mapped to a colon tissue specific functional interaction network. Several clustering algorithms run on the colon-specific network and the most significant sub-modules between the clusters were identified. The biomarkers were selected by filtering differentially expressed genes which also involve in significant biological processes and pathways. Biomarkers were also validated on two independent datasets based on their differential gene expressions. To the best of our knowledge, such a cascaded network analysis pipeline was implemented for the first time on a large collection of normal colon and polyp samples. We identified significant increases in TLR4 and MSX1 expressions as well as decrease in chemokine profiles with mostly pro-tumoral activities. These biomarkers might appear as both preventive targets and biomarkers for risk evaluation. As a result, this research proposes novel molecular markers that might be alternative to endoscopic approaches for diagnosis of adenomatous polyps.

## Introduction

Colorectal polyp is a protuberance into the lumen from the colonic mucosa. They are usually asymptomatic but ulceration, bleeding, tenesmus, and intestinal obstruction could be observed. Moreover, they could be either non-neoplastic (inflammatory, hamartomatous, or hyperplastic polyps) or neoplastic (adenomatous) in nature. Adenomatous polyps of the colon are the most prevalent neoplastic polyps. About 5–7% of the adenomas have high-grade dysplasia and 3–5% of the cases have invasive carcinoma at the time of diagnosis [1–3]. Even though the majority of these adenomas do not go to malign transformation called adenoma-to-carcinoma sequence, most of colorectal carcinomas originate from neoplastic polyps [4]. Therefore, insight on the transformation process could support both preventive therapies and biomarkers indicating the risk of malignancies.

The formation of neoplastic polyps depends on multiple cumulative mutations either activating oncogenes or repressing tumor suppressors. These genetic alterations are mostly related with proliferation, survival, and DNA repair mechanisms. In colon adenomatous polyp formation oncogenes such as K-RAS (Kirsten Rat Sarcoma Virus) and MYC as well as tumor suppressors such as APC (Adenomatous Polyposis Coli) and p53 are more common whereas sessile tumors present mutations in DNA repair genes MLH1 (MutL homolog 1) and MLH2 (MutL homolog 2) [5,6]. Since our research focused on adenomatous polyps, sessile serrated samples are excluded. Moreover, adenomatous polyps induce inflammation and immune response through secreting chemokines and presenting tumor associated antigens with HLA (human leukocyte antigen) family. Immune players such as macrophages and lymphocytes migrate tumor microenvironment with an intention to guide damaged cells to death. However, most tumors could reprogram immunity to more supportive phenotype [7,8]. Inflammation could also enhance mutation profile due to increased reactive oxygen species related radical damage [9]. As the first mutation initiate polyposis, genetic damage accumulates to advance the neoplasm. Although damaged cells frequently exhibit dysplasia, an adenoma could not penetrate basement membrane and metastasize. Nevertheless, there is a threshold where the dysregulated pathways lead more invasive and metastatic phenotype. This threshold defines the border between benign and malign tumors.

The majority of colon adenomas are asymptomatic. Therefore, routine screening is crucial in the diagnosis of polyps at early stages regarding the risk of malignancies. Despite its low specificity and sensitivity, Fecal Occult Blood testing is a less irritating and relatively cheap method to detect colon polyps. Yet, colonoscopy is the "gold standard" in diagnosis of colon lesions due to higher sensitivity. World Health Organization recommends this screening method every 5 years beginning at the age of 50. There are also some other endoscopic applications combined with immunocytochemistry to treat the polyp and diagnose its molecular profile [6,10]. However, the procedure is both irritating and intimidating for majority of patients. For this reason, newer applications of less irritating methods such as “Fecal immunochemical testing” and “Fecal DNA and antigen testing” emerged. These assays frequently target molecular biomarkers in stool [10]. Recently trending exosomes and liquid biopsies could also make it possible to diagnose adenomas and evaluate the risk of malignancy at early stages [11,12]. Thus, the need of biomarkers calls for further research to identify molecular risk factors and milestones in adenoma-to-carcinoma sequence.

This study presents alterations in several gene expressions as potential biomarkers of adenomatous polyps that were identified by a computational pipeline composed of several methods. The analysis performed on unified mRNA patient samples, which are obtained from eight different microarray studies covering normal colon and adenomatous polyp samples. The results identified increases in TLR4 (The tool-like receptor 4) and MSX1 (msh homeobox 1) expressions as well as decrease in chemokine profile with mostly pro-tumoral activities. These results were also validated on independent two cohorts. Therefore, these genes can emerge as preventive targets and biomarkers. In these regards, this research suggests molecular markers as alternative to endoscopic approaches.

### Background

This section briefly summarizes functions of biomarker genes, their roles in adenomatous polyp and cancer development, and computational approaches developed for colon polyp and colorectal cancer analysis.

#### The tool-like receptor (TLR) family

The TLR family is a bunch of pattern-recognizing membrane proteins which are crucial in pathogen detection and immune response. Ligands such as lipopolysaccharides, viral fusion proteins or bacterial glycolipids initiate the dimerization of them which eventually translocate the nuclear factor (NF)-κB into the nucleus and induces inflammatory mediators and response [13]. They do not only make function in macrophages and dendritic cells, but also take essential roles in the epithelia of the gastrointestinal tract [14]. Intestinal cells express them to detect gut microbiota and maintain epithelial cell integrity through tight junctions. They are also correlated with proliferation and differentiation. In addition to physiologic immune response, they are included in pathogenic processes as well. Recent studies indicate that the TLR family could take pivotal role in promotion of gastrointestinal malignancies. They have an impact on immune suppression, matrix dysregulation, and metastasis in colorectal tumors [15–17].

An infamous member of this family, TLR4 have been associated with colorectal cancers as well as polyp formation [18,19]. Several studies indicate higher levels of expression in villous/tubulovillous polyps and tubular adenomas along with colorectal cancers. TLR4 deficient mice were protected from colon carcinogenesis [19]. Moreover, the expression levels were correlated with F. nucleatum, E. faecalis, S. bovis. Therefore, TLR4 emerges as another mechanism of how dysregulated microbiota could affect either promotion or progression of colon malignancies [20]. Some treatment strategies antagonize them to hinder malign transformation and discuss their effectiveness over antibiotics [21]. Nonetheless, this immune regulator receptor not only modulates tumor-associated inflammation and immune suppression, but also cross-signals cancer-associated pathways such as EGFR, PI3K, VEGF, NF-κB [22–24].

#### MSX1

MSX1is a homeobox gene taking a role in developmental processes in various tissues during embryogenesis and morphogenesis. Previous studies indicate that it is mostly expressed in progenitors and is crucial in differentiation. Human tooth development, odontogenesis, is most-mentioned process correlated with this transcriptional factor [25]. Recently, accumulating number of researches identified this transcription factor as a tumor suppressor and biomarker for longer progression-free survival in some malignancies including glioblastoma, melanoma, lung, endometrial, ovarian, and cervical cancers [26–30]. Hypermethylation and loss of expression are often correlated with poor prognosis, metastasis, and drug resistance. Also, Bonito and colleagues suggest that it supports p21 regulated apoptosis [31]. Even though MSX1 hypermethylation and down-regulation are also reported in colon cancer, Horazna and colleagues point out that MSX1 is overexpressed in colon villous adenomas and takes a crucial role in tumor initiation due to APC loss [32–34]. The impact of this homeobox on colon cancer and the malign transformation is not entirely understood.

#### Chemokines

Directional migration of leukocytes is called chemotaxis. And chemotactic cytokines are chemokines. They are secreted by leukocytes, epithelial, endothelial, and tumor cells [35]. Cancer- or tumor-associated fibroblasts (CAFs/TAFs) also secrete tumor promoting CXC chemokine stromal cell-derived factor-1 (SDF-1)/CXCL12 [35].

Recently, it is shown that CXC chemokines and their receptors (CXCR) may affect tumor behavior. They have got a role in angiogenesis, leukocyte attraction, proliferation and metastasis. They have also autocrine or paracrine effects. In general, increased expression of CXC chemokines correlates with poor prognosis [35]. The N-terminal region contains cysteine residues and is subdivided into four families according to position of these residues: CXC, CC, C, and CX3C chemokines. X stands for any amino acid [35]. The presence of the tripeptide motif (Glu-Leu-Arg at the NH2 terminus) is also important for sub-classification: ELR+ or ELR- [36]. The chemoattraction of pro-tumoral or anti-tumoral leukocytes depends on the secretion of ELR+ and ELR- CXC chemokines, respectively [35].

In addition to the major findings of TLR4 and chemokines, there were various alterations including distinct pathways that are also determined and discussed in the results section.

#### Computational approaches

There are studies on different types of cancer in which one or more computational approaches such as co-expression network, differential expression gene analysis (DEG), pathway analysis, and protein-protein interaction (PPI) are applied. In a recurrent glioblastoma study, weighted gene co-expression analysis (WGCNA) and DEG analysis were used together on GEO data [37]. There are studies in which gene expression and pathway analysis are evaluated together with survival analysis for detection of target genes that may play a role in metastatic colon cancer [38]. In order to investigate the development of colon cancer, different gene expression analysis and PPI networks were used to identify important genes [39]. To identify biomarkers for diagnosis of colorectal cancer, a differential gene expression analysis was applied on TCGA and GEO datasets; important hub genes were detected by using the STRING network [40]. A similar study integrated mRNA expression data and a PPI network to identify important genes in the survival of colorectal cancer patients [41]. Some of the biomarkers were validated by real-time quantitative PCR analysis. A recent study applied WGCNA on colorectal cancer samples obtained from two GEO datasets [42]. The nodes with higher degrees in the network were identified as hub genes after mapping of differentially expressed genes found in important modules over the STRING network. For the differentiation of polyp subtypes, the gene expression differences between two different polyp groups were defined by several technologies [43]. In a meta-analysis study, normal colon, polyp and colon cancer gene expression data were integrated with the Combat method; DEG and pathway analysis were applied to explain the mechanisms of polyp and cancer formation [44]. In another study, DEG and pathway analysis were performed for normal, polyp and colon cancer samples; a marker gene cluster was revealed [45]. To understand molecular features of colorectal adenomas, cancer free and cancer adjacent polyp samples were collected, and various analysis were applied on genome, transcriptome and methylome data [46]. There is no study in the literature presenting the co-expression relationship between normal colon and colonic polyp tissue by applying co-expression network and clustering analysis together.

This study aimed to explain the reasons of molecular differences between normal colon and polyp tissue at the gene expression level by composing a larger patient cohort. We performed data integration by using batch effect removal on cohorts of different studies. The integrated data were analyzed by the WGCNA. This analysis led to highly correlated and significant modules for normal colon and polyp samples. The selected modules were further clustered to focus on functionally conserved proteins. By applying network clustering algorithms, larger co-expression modules became more targetable. As a final step, a gene enrichment analysis revealed biomarker genes with increased/decreased mRNA expression profiles. The identified biomarkers were validated on two independent datasets by applying differential expression analysis. Based on gene expression behavior, the potential drugs targeting selected biomarkers were also reported. Such integrated network-based approaches were used for analysis of a large collection of normal colon and polyp samples for the first time. Due to covering large number of patient samples, the identified biomarkers present higher statistical significance in terms of further clinical validations.

## Materials and methods

**Fig 1:**
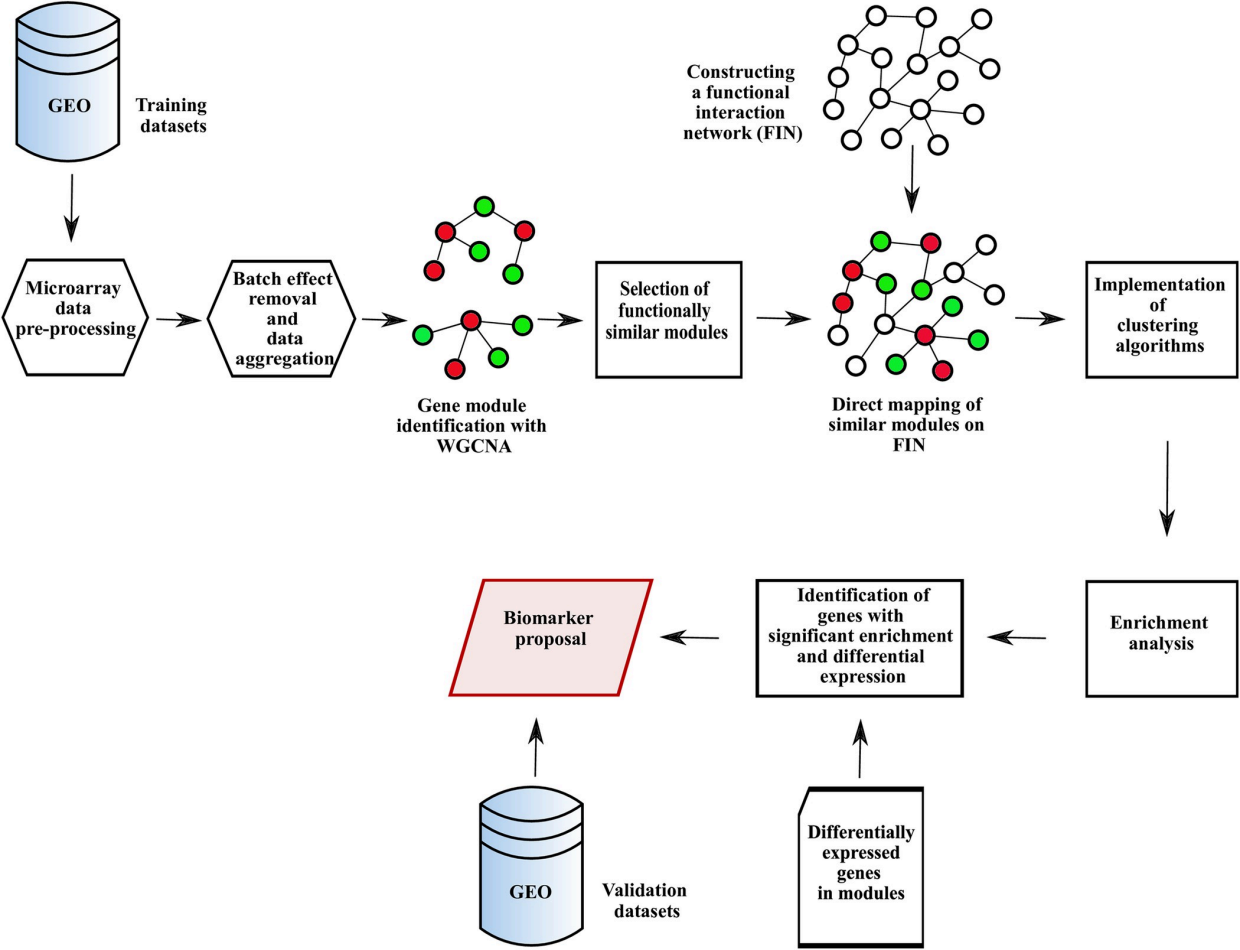
An overview of the study.

This section presents a description of the datasets and stages of the study. Fig 1 shows an overview of the study. Normal colon and polyp samples in the GEO database were used to identify biomarkers in polyp formation process. Various preprocessing operations, correction of batch effect and data aggregation were applied. The WGCNA was applied to identify gene modules, then modules with similar profiles were selected. By constructing a functional interaction network (FIN) specific to the colon tissue, modules with similar profiles obtained from the previous step were directly mapped to this network. Then, various clustering algorithms run on the obtained network and the most significant submodules between the clusters were identified. Enrichment analysis was applied for the selected significant submodules. As a result of this analysis, genes that are both involved in significant biological processes and pathways and differentially expressed between normal colon and polyp tissues were selected as biomarkers.

### Dataset selection

We filtered the Affymetrix hgu133plus2 chip experiments for normal colon and polyp tissues to use for training of a model and finally obtained eight datasets available in the GEO database. In addition, two data sets, one of them Affymetrix hgu133plus2 (GSE37364) and the other one Affymetrix hgu133a (GSE68468) chip experiment, were used for the validation of the initial model. The total number of patient samples of training and validation sets are given in Table 1.

**Table 1:** Datasets used in the study.

GEO Accession	Normal colon	Polyp	Training Set	Validation Set
GSE4107	10		**✔**	
GSE4183	8	15	**✔**	
GSE8671	32	32	**✔**	
GSE9348	12		**✔**	
GSE10714	3	5	**✔**	
GSE13471	4		**✔**	
GSE15960	6	6	**✔**	
GSE18105	17		**✔**	
GSE37364	38	29		**✔**
GSE68468	55	51		**✔**
**Training**	92	58		
**Validation**	93	80		
**Total**	185	138		

### Microarray data pre-processing

When the data sets for training were examined, it was seen that there were 604258 different probes at the beginning. The “rma” (Robust Multi-Array Average) method of the “affy” library in R-Bioconductor was used for the normalization of these probes. After the normalization process, 54675 probes remained in the data sets. These probes were annotated using the Entrez identifier of each gene. A total of 12753 probes were found to be labeled as “NA”, these probes were removed and a total of 41922 probes with gene name tags were found.

The same preprocessing method was applied for the validation sets. Since one of the validation sets was Affymetrix hgu133plus2 chip same as the ones in the training set, the number of probes obtained became the same. For the Affymetrix hgu133a microarray in the validation set, 22283 probes were obtained after applying the "rma" normalization. These probes were annotated using the Entrez identifier of each gene. A total of 2077 probes were found to be labeled as “NA”, these probes were removed and a total of 20206 probes with gene name tags were found.

### Batch effect elimination and data aggregation

A gene is represented by more than one probe in microarray chips. In order to aggregate several measurements of each gene, the median value of the repetitive probes was taken and assigned as the mRNA expression value of each gene. After this aggregation process, a total of 20174 probes representing individual gene regions remained for 150 samples in the training data set. In the validation set, total number of probe numbers remained the same for the Affymetrix hgu133plus2 microarray (GSE37364), while it was obtained as 12645 for the Affymetrix hgu133a microarray (GSE68468).

In order to obtain more statistically significant analysis, it is necessary to reduce the “batch” effect between gene expression data produced in different laboratories using the same platform—GPL570 (Affymetrix Human Genome U133 Plus 2.0 Array) in training sets. The "ComBat" function in the "sva" package in R-Bioconductor is used for this process. The dendrogram obtained by hierarchical clustering used to evaluate the results is given in S1 and S2 Figs.

Since the two experiments in the validation dataset belong to different platforms (GPL570 and GPL96), the batch effect removal method was not applicable on validation samples, so they analyzed independently by performing statistical significance tests.

### Network analysis

We used an integrated FIN with sufficient information about biological processes specifically related to cancer formation and progression [47]. Within this network structure, each node represents a human protein, and a link connecting two proteins represents a weight value that shows how biologically similar processes these two proteins work on. The FIN consists of 20790 proteins (nodes), 21952150 interactions (links). The weight value of each interaction represents the similarity of biological function between two proteins, and these values range from 0 to 1. Proteins with very low functional similarity (those with 0–0,1 range) were excluded. After this filtering, 15002 proteins and 334225 interactions remained in the FIN.

In order to eliminate the noise that may occur in the subsequent analyzes, we used the "Tissue Atlas" data within the "Human Protein Atlas" project. We eliminated the proteins, which are not synthesized in healthy colon tissue, and the connections between them from the FIN. We obtained a colon tissue specific interaction network. Technically, Entrez identifiers of the genes that are expressed in the colon tissue were obtained. As a result of this analysis, 14486 genes and 234189 common links were obtained for colon tissue. Then, the "components" function in the "igraph" library was used to determine the submodules on this colon tissue-specific network. 24 submodules were identified, the largest module covers 11355 genes and 234152 links. Subsequent analyzes continued with the largest module structure.

### Weighted gene co-expression analysis

Weighted gene co-expression analysis was performed with 20174 gene expression data of 150 patients in our training dataset using the WGCNA library in R-Bioconductor. Thus, it was aimed to extract the gene expression modules that have the highest correlations with given phenotypes of the patients.

First, the soft threshold power was selected according to the scale-free topology criterion [48]. Using this soft threshold value, a weighted gene adjacency matrix is defined that represents a gene co-expression network in which each link shows the co-expression similarity between a pair of genes. Then, the adjacency matrix was transformed into the topological overlap matrix (TOM) to minimize the effects of noise. The dissimilarity matrix was calculated by subtracting the TOM from 1. Hierarchical clustering of the difference matrix (with the "hclust" function) was used to identify the modules in the network. This function produces a clustering tree (dendrogram). When branches of the tree are densely interconnected, they represent modules formed by highly co-expressed genes. Detecting modules means identifying the branches of the tree by cutting them. The dynamic tree cutting method from the “dynamicTreeCut” package, which is a standard method for cutting branches, was used. Dynamic tree cutting may identify modules with very similar gene expression profiles. The genes of such modules were combined because they were highly co-expressed.

### Network-based clustering

The genes involved in each significant module were combined and directly mapped on the FIN, which was previously customized for colon tissue. The "ego" function in the "igraph" library is used for this filtering process. The network structure in which the genes in each module are directly adjacent to each other in the FIN was used. There are 478 genes and 2846 links directly connected to each other in the network obtained by this method.

In order to focus conserved network modules in terms of biological functions, we applied network clustering algorithms to specific modules selected as a result of WGCNA and mapped to FIN. Markov clustering (MCL), fuzzy neighborhood (FN) and spectral clustering algorithms were run separately on the same network to find the most relevant submodules. Marker genes that play an active role in polyp formation might be detected more effectively in this way.

The MCL algorithm runs using libraries in Python and R languages. Different values have been tested for the inflation and expansion parameters, which are the critical arguments of the MCL algorithm. Since the highest modularity score was obtained when the inflation operator was "1.2" and the expansion operator was "2", the algorithm runs with these values. For the fuzzy neighborhood algorithm, the "cluster" function in the “ProNet” package was used by setting the method parameter as "FN". For the spectral clustering algorithm, the "SpectralClustering" function in the Python "Sklearn" library was used. The algorithm runs with the following parameter values: affinity as "precomputed", assign_labels as "discretize", random_state as "0".

The performance of each clustering algorithm was evaluated using both internal and biological metrics. Internal evaluation metrics evaluate only on clustered data, without reference to externally provided results (such as cluster labels). The internal metrics used are modularity and silhouette.

Modularity, one of the most popular validation criteria for topological clustering, states that a good cluster should have more interior edges than expected and fewer inter-cluster edges than expected, compared to a random network with similar properties. The modularity score *Q* calculated for a clustering is given in Eq 1; where *m* is the number of sides; *A*_*ij*_ is an element of the neighborhood matrix *A* in row *i* and column *j*; *k*_*i*_ and *k*_*j*_ denote the degree of *i* and *j*, *c*_*i*_ and *c*_*j*_ are components of *i* and *j*, respectively. The sum is calculated for all pairs of vertices *i* and *j*; where δ(x,y) is taken as “1” if x = y, and “0” otherwise.


$$Q=\frac{1}{2m}\sum_{i,j}{(A}_{ij}-\frac{{k_{i}k}_{j}}{2m}\delta (c_{i},c_{j})$$ (1)

The "modularity.igraph" function from the "igraph" library was used to calculate the modularity of a clustering. The higher the modularity score Q value, the better the topological clustering [49].

The silhouette index *S(u)* represents the average of the silhouette value *S(i)* of each observation. *S(i)* was calculated using the mean intra-cluster distance (a) and the mean nearest-cluster distance (b) for each sample (Eq 2). The *S(i)* lies in the range of [–1,1]; well clustered observations have values close to 1 and vice-versa. The “index.S” function in the “clusterSim” library was used to calculate the silhouette index score [50].


$$S(i)=\frac{b(i)-a(i)}{\left(\max\{(a(i),b(i))\right\}}\;\mathrm{where},\;S(u)=\sum_{i=1}^{n}S(i)/n$$ (2)

Biological metrics evaluate the ability of a clustering algorithm to generate biologically meaningful subsets. The biological metrics used in this study are the Biological Homogeneity Index (BHI), Wang Biological Process (BP), and Molecular Function (MF) Index.

BHI measures how biologically homogeneous clusters are. *B = {B*_*1*_,*…*., *B*_*F*_*}* is defined as an *F* functional class sequence, *B(i)* is defined as the functional class containing gene *i*, *B(j)* is defined as the functional class containing gene *j*, and *I(B(i) = B(j))* takes the value “1” if *B(i)* and *B(j)* are the same, “0” otherwise. Genes placed in the same cluster are assumed to have the same biological functions. For a given clustering segment *C = {C*_*1*_,*…*.,*C*_*K*_*}* and a biological class sequence *B*, the BHI value is given in Eq 3 [51]. Here, *n*_*k*_ *= n(C*_*k*_*/B)* and *C*_*k*_ is the number of genes in the statistical set. The BHI value is in the [0,1] range, the larger values corresponding to more biologically homogeneous clusters. The “BHI” function in the “clValid” library was used to calculate the BHI score.


$$BHI(C,B)=\frac{1}{K}\sum_{k=1}^{K}\frac{1}{n_{k}(n_{k}-1)}\sum_{i\neq j\in C_{k}}I(B(i)=B(j))$$ (3)

Wang *et al*., used the semantic similarity between gene ontology (GO) terms to calculate how functionally similar the genes in the detected clusters were [52]. They used a network-based method that uses the topology of the GO mesh structure to calculate semantic similarity. The semantic similarity of two GO terms is determined based on both their positions in the GO hierarchy and their relationship to ancestor terms in the network. In this method, the semantics of GO terms are encoded in a numerical format and different semantic contributions of different relations are considered [52]. The Wang Biological Process (BP) and Molecular Function (MF) Index were calculated with the “mgeneSim” function in the “GoSemSim” library.

Furthermore, significant gene expression analysis was performed to show gene expression changes between the normal colon tissue and the polyp in the modules obtained by WGCNA. In this analysis, both student’s t-test and fold change were calculated. The *p-*values were corrected using the "False positive rate (FDR)" method. Statistically significant gene lists were obtained by filtering genes with absolute fold change value > 1.0 and FDR < 0.05. Then, the common genes between selected WGCNA modules and statistically significant ones were identified by comparing colon tissue and polyp samples.

Considering both internal and biological evaluation metrics, the submodules determined by the clustering algorithms that provide the optimum clustering results were re-evaluated with the individual BHI, Wang-BP, Wang-MF criteria, and finally the submodules with the highest biological evaluation criteria were selected. Then, significantly expressed genes within these submodules were selected for biomarker analysis.

### Enrichment analysis

The GO biological processes and KEGG pathways covering the genes in the significant modules were determined using the enrichR software [53]. Terms with a 0,05 or lower *p-*values (FDR adjusted) were selected as significant.

The pathway results were visualized by using the term_gene_graph (edited) function in the pathfindR package to summarize gene expression changes and pathways memberships [54].

### Validation analysis

After applying data preprocessing and probe aggregation operations on experiments (GSE37364, GSE68468) in the validation data set, differential expression analysis was applied to each experiment separately. We aimed to identify the differentially expressed genes that were statistically significant between normal colon tissue and polyp for each data set. In this analysis, both the student’s t-test and the fold change value were calculated similar to the training set. The *p*-values were adjusted by using the FDR method. Differentially expressed gene lists were obtained by filtering genes with absolute fold change > 1.0 and FDR < 0.05. Differentially expressed genes for validation sets were compared with the genes found in significant submodules that were identified by the network analysis of the training set.

## Results

We summarize the results of computational analysis pipeline in this section.

### Gene co-expression analysis

**Fig 2:**
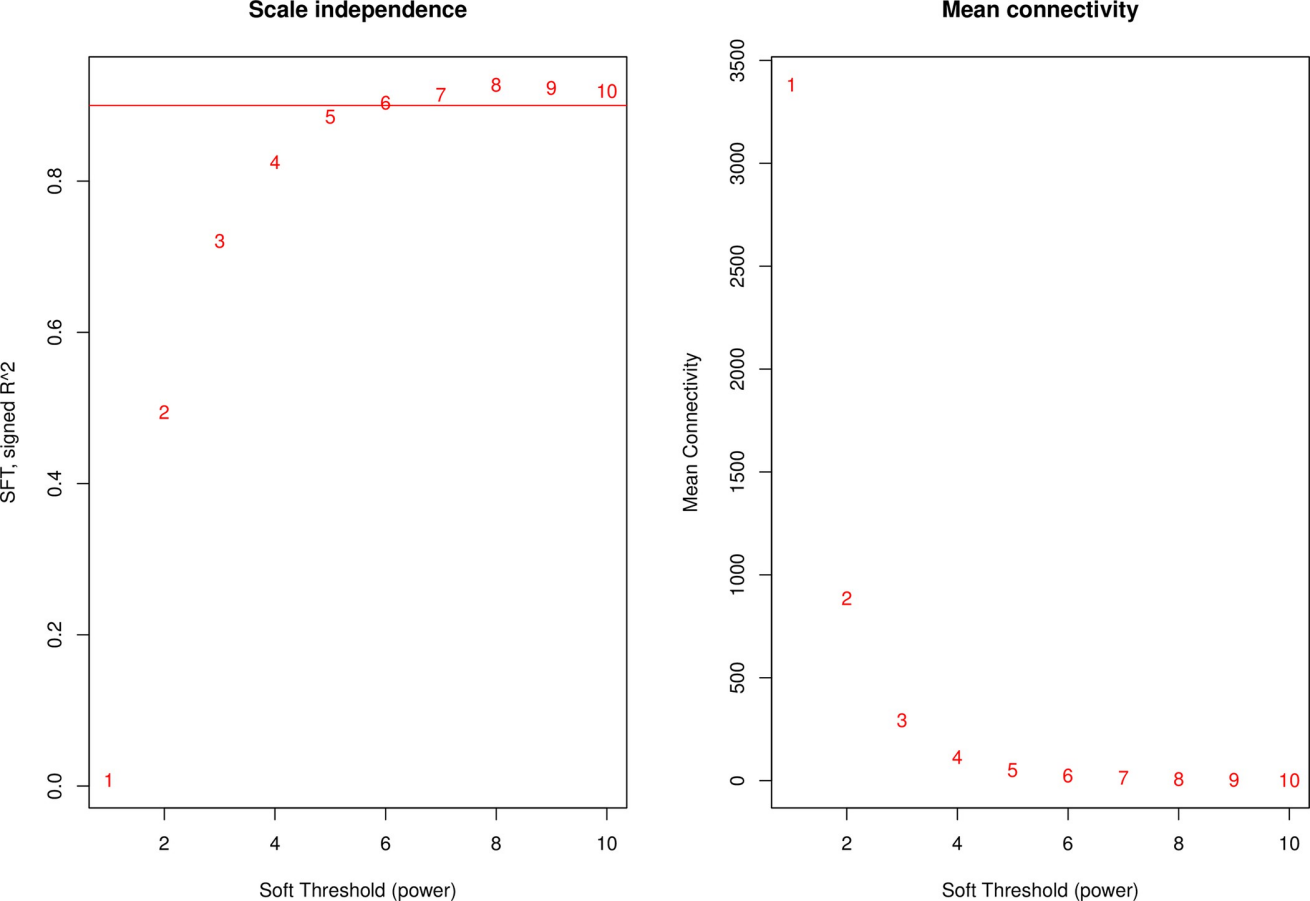
Network topology analysis for various soft thresholds. The left panel shows the scale-free fit index (y-axis) as a function of the soft threshold value (x-axis); the right panel shows the average connectivity (y-axis) as a function of the soft threshold value (x-axis).

WGCNA creates a gene co-expression network in accordance with the scale-free topology criterion [48]. As shown in Fig 2, the lowest threshold value of “8” was chosen when the scale-free topology fit index curve reaches a high value and flattens out.

**Fig 3:**
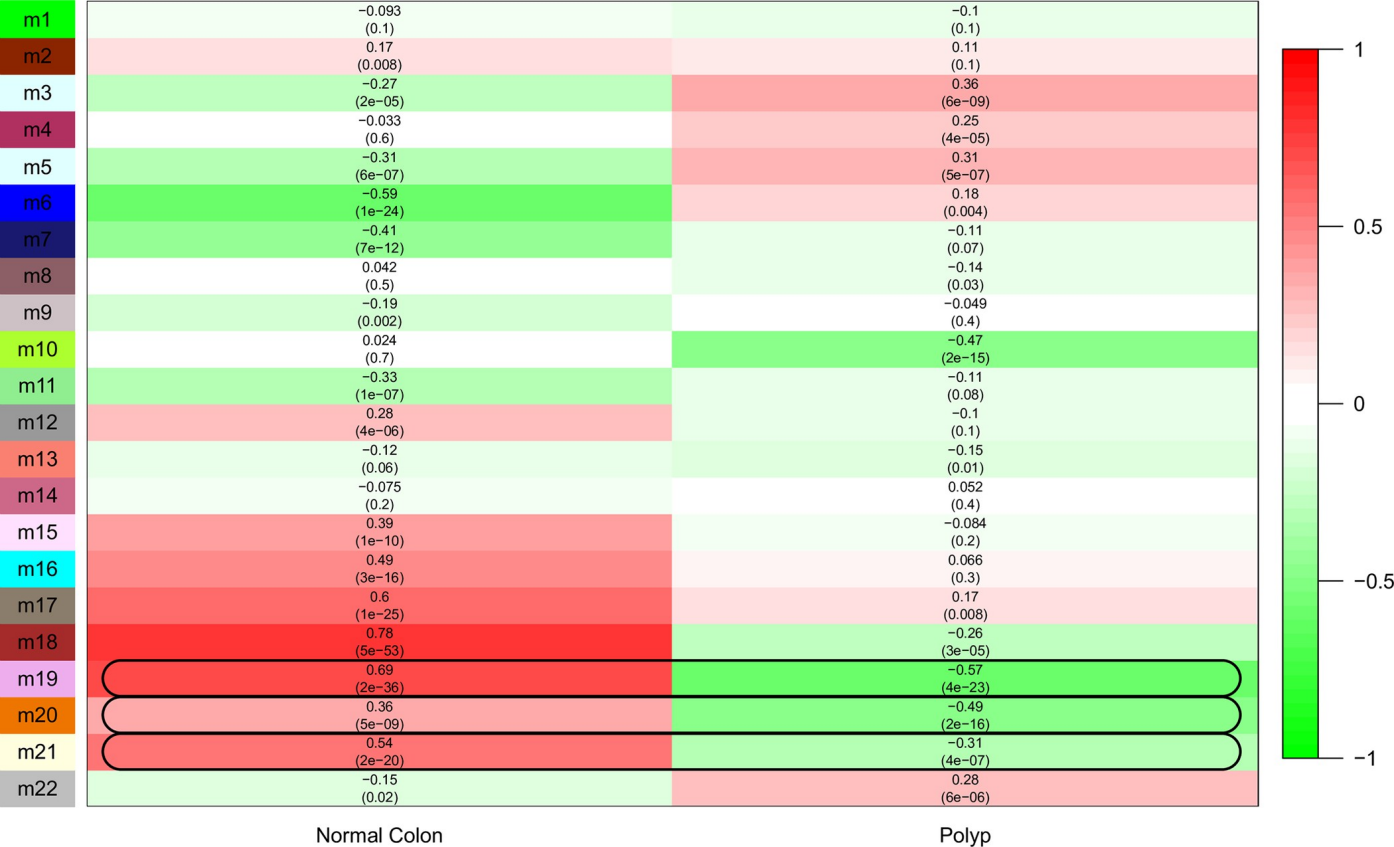
Correlation matrix resulting from WGCNA applied on the training dataset. Here, each cell indicates the Pearson correlation and *p-*value resulting from the association between the respective module eigengenes (row) and phenotype (column).

Fig 3 shows a correlation matrix resulting after applying WGCNA on the training dataset. Dynamic tree cutting was used to identify modules with very similar gene expression profiles. Therefore, 0.25 height cut was set as the threshold, which corresponds to a correlation of 0.75. When similar genes were combined based on the threshold value, we obtained 22 modules.

When we analyzed *p-*values and correlation values, only three modules were extracted that showed a relatively significant correlation (>0.3) between the normal colon and polyp samples. These modules have opposite expression patterns for normal colon and polyp, i.e., the module genes are upregulated in normal colon, same genes downregulated polyp samples. The total number of genes in these significant modules are given in Table 2.

**Table 2:** Selected significant modules and the number of genes obtained on the dataset.

Module number	Number of genes in the module
m19	205
m20	517
m21	35
**Total**	**757**

### Network clustering and identified submodules

The genes in the three significant modules of the training dataset (m19, m20, m21 marked in Fig 3) were matched on FIN, and the resulting network structures were given as input to different network clustering algorithms (MCL, FN, Spectral). The performance of each algorithm was evaluated by using both internal and biological metrics, these results are summarized in Table 3.

**Table 3:** Performance comparison of clustering algorithms.

Evaluation Metric	MCL(R)	FN	Spectral	MCL(Python)
Modularity	0.146	0.364	0.327	0.213
Silhouette	0.004	0.023	0.019	0.021
Average_BHI	0.251	0.283	0.262	0.200
Average_WangBP	0.471	0.479	0.461	0.362
Average_WangMF	0.571	0.617	0.596	0.411

Considering both internal and biological evaluation metrics, it was seen that the FN and Spectral algorithms generally provided better clustering results. The submodules detected by these algorithms were re-evaluated with individual BHI, Wang-BP Wang-MF metrics. First of all, submodules with the highest BHI, Wang-BP, Wang-MF values were selected, then the presence of differentially expressed genes in the relevant submodule was considered.

The annotated biological processes and pathways of genes found in 15 different submodules were determined by applying an enrichment analysis; the results are listed in Table 4. As a result of these analyzes, submodules 3, 9 of the FN algorithm and submodule 1 of the spectral algorithm were found to be biologically more significant.

**Table 4:** Summary of significant submodules detected by the best performing FN and Spectral clustering algorithms.

Clustering Algorithm	Submodule No	Number of Genes	BHI score	Wang_BP	Wang_MF	Number of downregulated genes	Number of upregulated genes
FN	2	116	0.317	0.337	0.654	14	0
	3	92	0.158	0.299	0.475	32	1
	4	39	0.083	0.246	0.406	14	1
	5	29	0.393	0.441	0.697	5	0
	7	43	0.161	0.308	0.526	8	1
	9	17	0.367	0.569	0.741	2	0
	10	11	0.427	0.718	0.849	2	0
	24	4	0.500	0.740	0.779	0	0
	1	128	0.307	0.333	0.624	20	1
Spectral	6	7	0.500	0.701	0.724	2	0
	12	13	0.436	0.694	0.844	3	0
	14	7	0.500	0.559	0.726	0	0
	19	18	0.422	0.449	0.788	3	0
	20	38	0.100	0.311	0.557	10	0
	28	44	0.209	0.263	0.463	21	1

### Enrichment analysis

An enrichment analysis was applied to the selected three submodules (3^rd^ and 9^th^ submodules of FN, 1^st^ submodule of Spectral algorithm). In order to summarize enriched terms and related genes with a visual presentation, we drew a network plot in which nodes are either genes or enriched terms (KEGG pathway, GO biological process, cancer hallmark term). The genes are colored based on up-regulation (red) or down-regulation (green) expression behavior for polyp samples.

**Fig 4:**
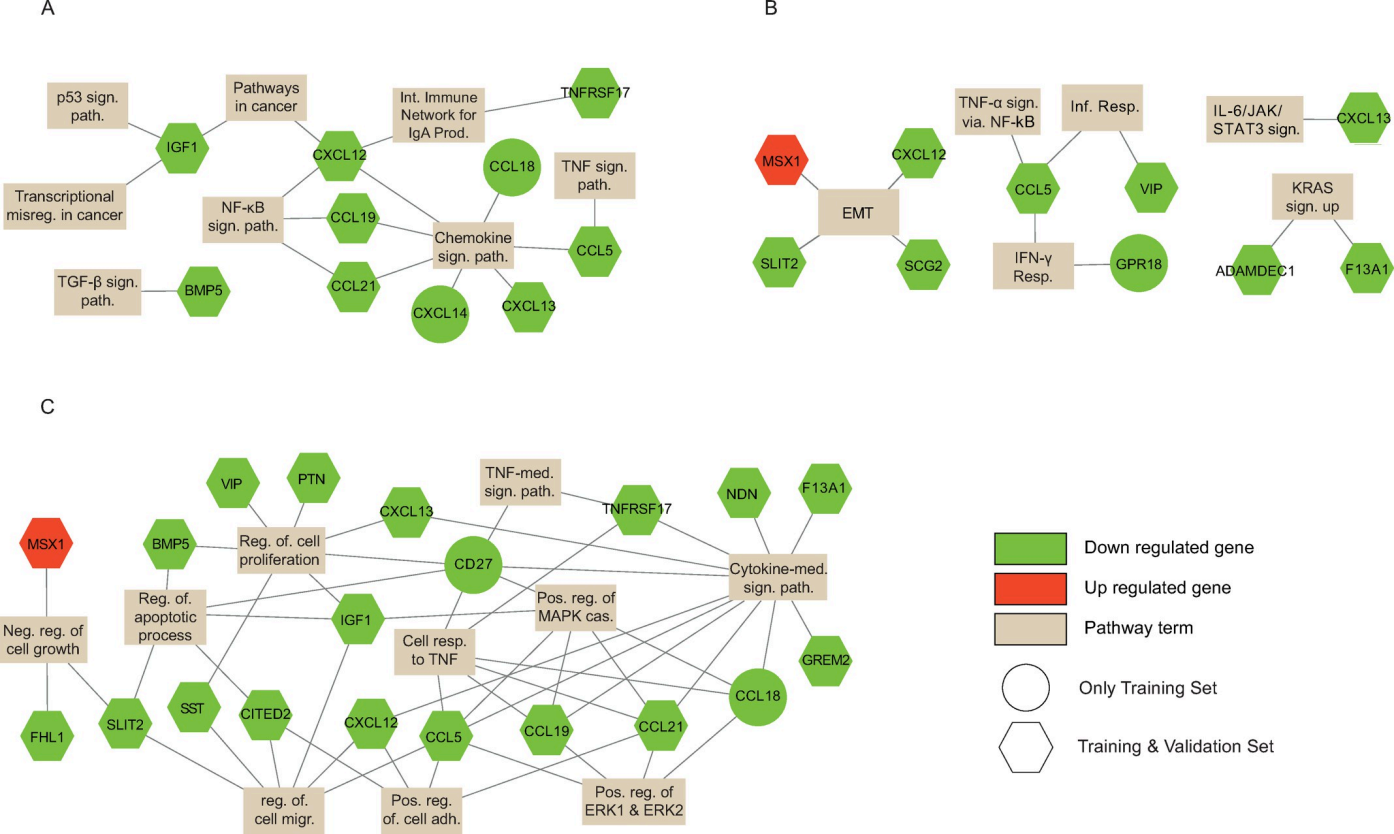
The gene-term graph in which the up / down regulated genes of the terms that are significant terms (A. KEGG pathway, B. Cancer Hallmark term, and C. GO-BP) for the 3^rd^ module of the FN algorithm.

The significantly enriched terms for the 3^rd^ submodule of the FN algorithm are presented in Fig 4. Most genes show a down-regulated expression (32 genes) in polyp samples. The only one exception is the MSX1 gene which has an up-regulated expression. The significant KEGG pathways are chemokine signaling pathway (CCL5, CCL18, CCL19, CCL21, CXCL12, CXCL14, CXCL13), ​​NF-kappa B signaling pathway (CCL19, CCL21, CXCL12), intestinal immune network for IgA production (TNFRSF17, CXCL12), pathways in cancer (IGF1, CXCL12). Some of the significant GO biological processes are cytokine-mediated signaling pathway (TNFRSF17, CD27, F13A1, NDN, CCL5, CCL18, CCL19, CCL21, CXCL12, CXCL13, GREM2), cellular response to tumor necrosis factor (TNFRSF17, CD27, CCL5, CCL18, CCL19, CCL21), negative regulation of cell growth (MSX1, FHL1, SLIT2), positive regulation of MAPK cascade (CD27, IGF1, CCL5, CCL18, CCL19, CCL21), positive regulation of cell adhesion (CCL5, CCL21, CXCL12, CITED2), regulation of cell proliferation (BMP5, CD27, IGF1, PTN, SST, VIP, CXCL13). Some of the significant cancer hallmark terms are epithelial-mesenchymal transition (MSX1, CXCL12, SCG2, SLIT2), KRAS signaling up (F13A1, ADAMDEC1), inflammatory response (CCL5, VIP), IL-6/JAK/STAT3 signaling (CXCL13).

**Fig 5:**
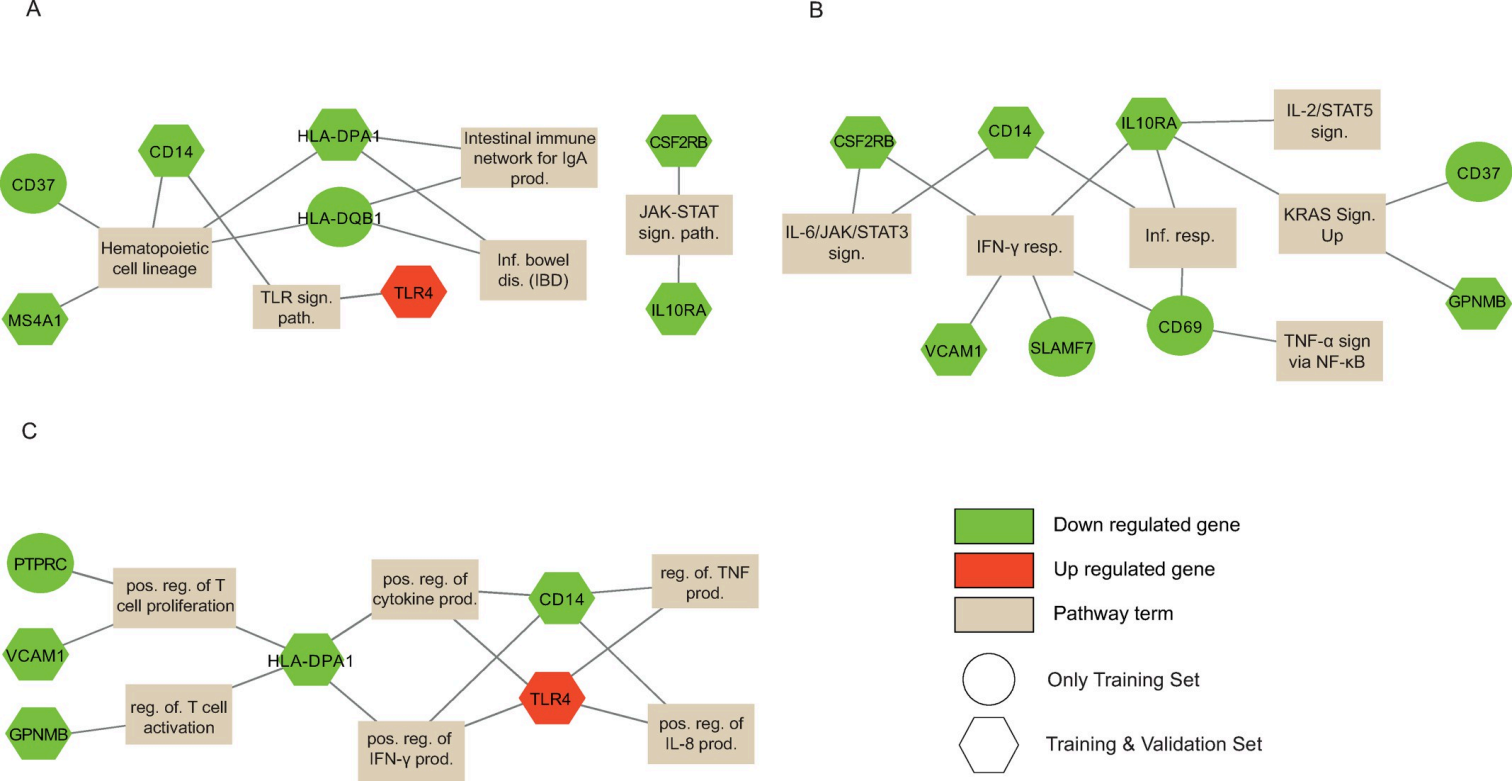
The gene-term graph in which the up / down regulated genes of the terms that are significant terms (A. KEGG pathway, B. Cancer Hallmark term, and C. GO-BP) for the 1^st^ module of the Spectral algorithm.

The significantly enriched terms for the 1^st^ submodule of the Spectral algorithm are presented in Fig 5. Most genes show a down-regulated expression (20 genes) in polyp samples. The only one exception is the TLR4 gene which has an up-regulated expression. The significant KEGG pathways are hematopoietic cell lineage (CD14, MS4A1, CD37, HLA-DPA1, HLA-DQB1), inflammatory bowel disease (TLR4, HLA-DPA1, HLA-DQB1), intestinal immune network for IgA production (HLA-DPA1, HLA-DQB1), JAK-STAT signaling pathway (CSF2RB, IL10RA), toll-like receptor signaling pathway (TLR4, CD14). Some of the significant GO biological processes are positive regulation of T cell proliferation (HLA-DPA1, PTPRC, VCAM1), positive regulation of cytokine production (TLR4, CD14, HLA-DPA1), positive regulation of interleukin-8 production (TLR4, CD14). Some of the significant cancer hallmark terms are inflammatory response (CD14, CD69, IL10RA), KRAS signaling up (CD37, IL10RA, GPNMB), IL-6/JAK/STAT3 signaling (CD14, CSF2RB), Interferon Gamma Response (CD69, CSF2RB, IL10RA, VCAM1, SLAMF7), TNF-alpha Signaling via NF-kB (CD69).

When we analyzed the significant pathway and terms associated with the differentially expressed genes of two submodules, the immune response and cytokine-mediated processes / pathways became more apparent out of all other enriched terms. Therefore, we will continue with the genes, which are members of these processes or pathways, in the biomarker identification procedure.

### Independent validation of biomarker proteins

As a validation procedure we compared the genes covered in three significant submodules (3^rd^ and 9^th^ submodules of FN clustering, 1^st^ submodule of spectral clustering) of the training set with the genes obtained as the result of differential expression analysis of the validation sets. In differential expression analysis of validation sets, there were 391 down-regulated and 236 up-regulated genes for the GSE37364 set, while 286 down-regulated and 77 up-regulated genes were found for the GSE68468 set.

When we compared the GSE37364 experiment with the 3^rd^ submodule of the FN clustering, most of the genes (25 genes) showed a down-regulated expression for both training and validation datasets (S1 Table). 17 of these genes (ADAMDEC1, BMP5, CCL19, CCL21, CCL5, CITED2, CXCL12, CXCL13, F13A1, GREM2, IGF1, NDN, PTN, SCG2, SLIT2, SST, TNFRSF17) are involved in significant biological processes as identified in the enrichment analysis. MSX1 has up-regulated expression in both datasets. Comparing the GSE37364 experiment with the 9^th^ submodule of FN revealed the down-regulation of CSF2RB and IL10RA in both training and validation set. In addition, these genes are involved in significant biological processes and pathways based on enrichment analysis. On the other hand, the comparison of GSE37364 experiment with the 1^st^ submodule of the spectral clustering led 13 common genes with a down-regulated expression in both data sets. 8 of these genes (GPNMB, HLA-DPA1, MS4A1, NDN, VCAM1, CD14, CSF2RB, IL10RA) are involved in significant biological processes. TLR4 has up-regulated expression in both datasets. CD14 has a down-regulated expression in both datasets.

When we compared the GSE68468 experiment with the 3^rd^ and 9^th^ submodules of the FN, all of the common genes showed a down-regulated expression in both datasets (S2 Table). 13 of these genes (ADAMDEC1, CCL19, CCL21, CITED2, CXCL12, CXCL13, F13A1, FHL1, GREM2, NDN, SLIT2, SST, VIP) are involved in significant biological processes. The comparison of GSE68468 experiment with the 1^st^ submodule of the spectral clustering showed 6 common genes with down-regulated expression profiles for both datasets. Two genes (GPNMB, NDN) are involved in significant biological processes.

As a summary, correlated results found between training and validation datasets confirm the consistency of biomarker proteins identified by the integrated network-based analysis of this study.

### Targeting biomarker proteins

We performed a computational drug screening which searched the known compounds targeting biomarker proteins identified in this study. The analysis was performed using the R package (“rDGIdb”) of the DGIdb system, which integrates more than twenty compound-protein interaction databases [55]. The compound groups with an inhibitory action were used for targets with significantly increased gene expression. The compound groups with an activator action were used for targets with a significant decrease in expression. Based on this compound action type filtering, we identified seven compounds for our biomarker proteins (Table 5).

**Table 5:** The results of drug screening that targets biomarker proteins identified a result of clustering analysis. The expression type column shows gene expression change of the gene. The action type was chosen according to the expression status of a target.

Target	Expression Type	Compound	Action Type	Compound Description
GPR18	Down	Arachidonoyl Glycine	Agonist	Endogenous agonist
	Down	Cannabidiol	Agonist	Active cannabinoid used as an adjunctive treatment
	Down	Anandamide	Agonist	
CSF2RB	Down	Sargramostim	Agonist	Immunostimulator for white blood cells as a chemotherapy drug
IL10RA	Down	Interleukin-10	Agonist	Anti-inflammatory cytokine
TLR4	Up	Resatorvid	Antagonist	Suppresses production of inflammatory mediators
	Up	Eritoran Tetrasodium	Antagonist	Toll-like receptor 4 inhibitor.

## Discussion

There is no identical study in literature that applies both WGCNA and tissue-specific network clustering on adenomatous polyp samples so far. Therefore, we compare our results with recent studies that perform various bioinformatics analysis on polyp and colorectal cancer samples.

Meng and colleagues applied WGCNA to identify hub genes in the progression of colorectal cancer [42]. They reported IL10RA as one of hub genes with a lower mRNA expression in colorectal cancer samples compared to normal. This result is correlated with our finding in which IL10RA also showed a decrease in polyp samples. Another study incorporated in silico and in vitro methods and proposed several potential therapeutic targets for colorectal cancer [41]. One of these targets was SST with a down-regulated expression in colorectal cancer samples, this result was also observed in our study. So, we speculate that some of the collected polyp samples in this study would present malignant profiles. Differential expression and PPI network analysis also revealed SST that was one of the genes with the highest AUC value in terms of diagnostic efficiency in colorectal cancer [40]. The VIP and SST genes reported in another study that applied network-based algorithms to find prognostic markers in colorectal cancer [39]. A decreased mRNA expression of VIP in colorectal samples was mentioned, which is also in parallel with our results since we observed down-regulated profile of VIP in polyp samples. Another study highlighted IGF1 as one of the drivers in the transition between adenoma and colorectal cancer, IGF1 was also identified in our study with a decreased expression profile in polyps [44]. Comparison of cancer free and cancer adjacent polyp samples presented several molecular changes between two polyp types [46]. In terms of mRNA expression, GREM1, IGF2, CTGF, and PLAU showed significant changes between cancer adjacent and cancer free polyps. There are common genes (BMP5, CCL18, MS4A1, MSX1, SCG2, SST, STMN2, VIP) which are differentially expressed in our study as well. Only BMP5 and STMN2 has the same expression behavior (down-regulation) with this previous study. Other common genes presented a reverse expression (down-regulation) in our study, this might indicate that some of the collected polyp samples in this study have more similar molecular profile to cancer adjacent polyps.

Almost all the genes analyzed in this study revealed decreased gene expression in polyp samples except for TLR4 and MSX1. Since these targets have been associated with several cancers, they could be promising biomarkers in adenoma-to-carcinoma sequence. Moreover, enrichment analysis points out their possible roles in either proliferation or invasion processes. Along with TLR4 expression, the results also indicate alterations in immune response regarding increased expression of MHC-II class genes and a shift in CD14 and chemokine profiles.

The results demonstrated that expressions of inflammatory bowel disease-related genes are significantly altered in colon polyp. Considering chronic inflammation is a well-known mechanism in carcinogenesis, regulation of these genes could be potential biomarkers and therapeutic targets in a view of treating adenomas and prevention [9]. One such gene, TLR4 is a major player in innate immunity and several studies correlated its role with colorectal carcinoma earlier [56]. Our results indicate its upregulation also in colon polyps against normal tissue, supporting its role in carcinogenesis. Recently, the function of this gene on promotion of adenomas related with various pathways such as NF-κβ/STAT axis and NOTCH signaling [34,57–60]. Also, gut microbiota and dysregulated immune response emerge as an essential mechanism [20,58,61]. A striking example has been given by Tsoi and colleagues. They indicate that a bacteria strain enriched in colon malignancies called Peptostreptococcusanaerobius induce proliferation through TLR4 related ROS activity [61]. Similarly, Pastille *et al*. demonstrated that blocking of TLR4 signaling abates progression of colitis-associated colon cancer progression through reduced pro-inflammatory response [21]. They claimed that this blocking treatment is superior to antibiotics during inflammatory phase due to observing less adverse effects. Interestingly, CD14 a co-receptor of TLR4 is downregulated in our study, contrary to TLR4 expression in adenoma. This result hints CD14-independent TLR4 function in colon polyp progression. Moreover, CD14 have been demonstrated as essential in macrophage polarization in response to LPS and IL-4 co-stimulation [62]. Considering inhibition of M2 polarization is CD14 dependent, downregulation of CD14 reprograms macrophages to M2 polarization which could support malign transformation and tumor associated microenvironment in colon adenomas through immune evasion and cytokine profile.

The pro-tumoral or anti-tumoral activities of chemokines previously reported which may be also necessary for polyp formation [35]. The colon polyps demonstrated decreased expressions in CCL5, CCL18, CCL19, CCL21, CXCL12, CXCL14 and CXCL13 genes in this research. These chemoattractant cytokines play crucial role in recruitment of immune cells into tumor microenvironment and regulation of inflammatory response. The downregulated chemokines are mostly related with pro-tumoral activities. Several studies indicate them as a poor prognostic marker. However, these immune regulators could also support immunity in some cancers. Similarly, decrease in CCL5, CCL21 and CXCL12 chemokines are related with reduced cell adhesion which could enhance invasive character for polyp [63–65]. Also, receptor profile on both tumor and immune cells manages the response. In these regards, the downregulated chemokines could be associated with either tumor suppression or progression. But decoy receptors and dysregulated tumorigenic signals could support polyp progression due to chemokines’ dual nature before the stage of malign transformation.

HLA-DPA1 and HLA-DQB1 genes take essential role in immunity. These two genes encode MHC Class II proteins over antigen presenting cells such as macrophages and dendritic cells, that present foreign or aberrant peptides to T cells. Downregulation of these genes could be part of immune evasion mechanisms. Several studies suggest that absence of HLA-DPA1 and HLA-DQB1 are correlated with poor prognosis of some cancers [66–69]. Low expression of HLA-DPA1 is associated with pediatric adrenocortical tumors [70]. On the other hand, HLA-DQB1 is more likely associated with autoimmune diseases [71,72]. There are limited studies on relationship of HLA-DQB1 with adenomas or malignities [68,73]. Zhang and colleagues indicate that this gene is a favored prognostic marker for early-stage lung adenocarcinomas [68]. Our results point out that both HLA-DPA1 and HL-DQB1 declined in colon adenomas. Therefore, we suggest that these two MHC class II family genes might contribute immune evasion in pre-malignant stage of colon tumors.

MSX1 takes role in infamous metastatic step called Epithelial Mesenchymal Transition (EMT) which is also defined as a cancer hallmark [9]. The process refers to a cellular differentiation in which epithelial cells gain mesenchymal features such as stem-like morphology, loss of cell polarity, increased migration, and invasion abilities [74]. Since MSX1 advances the invasive phenotype due to EMT, the upregulation of this gene could be beneficial in tumor progression. Moreover, the results point out that downregulation of SCG2, CXCL12, and SLIT2 genes are also correlated to this reprogramming behavior. Therefore, the expression changes in favor of EMT support the idea that colon adenomas could initiate to advance both invasive and metastatic features in pre-malignant period. Several studies endorse this proposition as they also discuss that EMT related pathways would involve in polyp formation [44,68,75]. Enrichment analysis illuminates that MSX1 upregulation, on one hand, induces EMT, on the other hand, hinders cell growth. This homeobox protein emerges as a tumor suppressor in various tumors such as breast, cervical, and glioblastomas. It inhibits tumor growth by suppressing proliferation signals and motivates cells to apoptosis [28,29,76]. Tao and colleagues also suggest this transcriptional factor inhibits migration and invasion in gliomas through the WNT / β-catenin pathway [29]. Nonetheless, several other studies conclude MSX1 as an onco-driver associated with increased invasion capacity and malignant phenotype [32,77,78]. Sun *et al*. indicated this factor as potential biomarker and therapeutic target for colorectal cancers [32]. Therefore, we could discuss that MSX1 might increase the response to over proliferative signal in colon adenomas as a brake mechanism, yet it could support invasive and metastatic phenotype to favor malign transformation given by its dual nature. If so, this transcriptional factor emerges as potential regulator of colon malignancies.

KRAS signaling related CD37, IL10RA, GPNMB had decreased gene expression patterns in polyp samples. GPNMB may be involved in growth delay and reduction of metastatic potential. A significantly higher methylation rate was found for the GPNMB gene in African American patients compared to Iranians [79]. The gene might play a role in the high incidence and aggressiveness of colorectal cancer in the African American population. The hypermethylation of the GPNMB gene is proposed as a marker of colon carcinogenesis. Another study showed that higher methylation profile of GPNMB led to a lower expression in adenoma and colorectal cancer samples [80]. So, the loss of GPNMB expression may cause tissue disruption and more invasive cells. This evidence is correlated with our observation, its expression loss might fasten the development of malignant polyps. IL10RA showed higher expression in healthy colon samples compared to colorectal cancer [81]. They claimed that IL10RA regulates immune system response in cancer environments. The loss of IL10RA expression in our results also supports limited immune response which can eventually cause the malignant profile of polyps.

## Conclusions

This study has analyzed 150 samples collected from eight different GEO projects covering both normal colon and adenomatous polyp tissue samples. In-silico pipeline was created with a holistic approach that applies co-expression analysis, tissue-specific PPI construction, network clustering, and pathway analysis. By integrating all these network-based approaches, we aimed to find molecular-level evidence of why the tissue transitioned to the polyp state before it became malignant. To the best of our knowledge, such a comprehensive network-based analysis was applied for the first time on colon polyp samples.

Construction of tissue-specific interaction networks have introduced a system level representation of real colon tissue to our computational model. Since the proteins without any expression in colon tissue were eliminated from the original PPI network, eventually only expressed ones remained in the colon-specific network. Usage of clustering methods on the colon-specific network revealed functionally conserved protein modules which were prioritized based on biological evaluation metrics. Finally, this analysis strategy highlighted biomarker proteins with highly similar molecular functions. Two independent cohorts also validated most of the biomarkers with similar differential gene expressions. As a summary, integration of systems biology methods and tissue-specific protein interactions revealed more significant biomarkers that can facilitate diagnosis and treatment of colon polyps.

The most significant biomarkers are TLR4 and MSX1 with upregulated expressions as well as several chemokines with downregulated expressions. TLR4 can be targeted by two compounds identified in a database search. Some of these genes take crucial role on EMT program and regulation of adhesion which induce more invasive and pre-metastatic phenotype to colon polyps even though they also play tumor suppressors as they hinder cell proliferation and tumor growth in pre-malignant stage. Moreover, TLR4 and aberrant chemokine profile indicate that inflammatory and immune mechanisms involved in polyp formation. Although these variances induce mostly anti-tumor response, a wide range of dysregulated survival signals and absence of regulatory proteins such as CD14 support tumor progression through M2 polarization and invasiveness. The invasive character could eventually favor malign transformation. Therefore, these alterations emerge as potential prognostic markers which could reveal malignant potential in adenomas. As a future work these findings will be confirmed by wet lab experiments to enlighten the roles of these targets as well as further investigations on inflammatory mechanisms and EMT programs in adenoma-to-carcinoma sequence.

## Supporting information

S1 Fig Hierarchical clustering result on 10 data sets without batch effect removed. (TIF)

S2 Fig Hierarchical clustering result on 10 datasets whose batch effect was removed by combat method. (TIF)

S1 Table Comparison of GSE37364 in validation set and significant submodules in training set. (DOCX)

S2 Table Comparison of GSE68468 in validation set and significant submodules in training set. (DOCX)
