# Supplementary figures and images for: In silico identification of novel biomarkers for key players in transition from normal colon tissue to adenomatous polyps
Source: PLoS One. 2022 Apr 29;17(4):e0267973. doi: 10.1371/journal.pone.0267973 (PMC9053805; doi:10.1371/journal.pone.0267973)

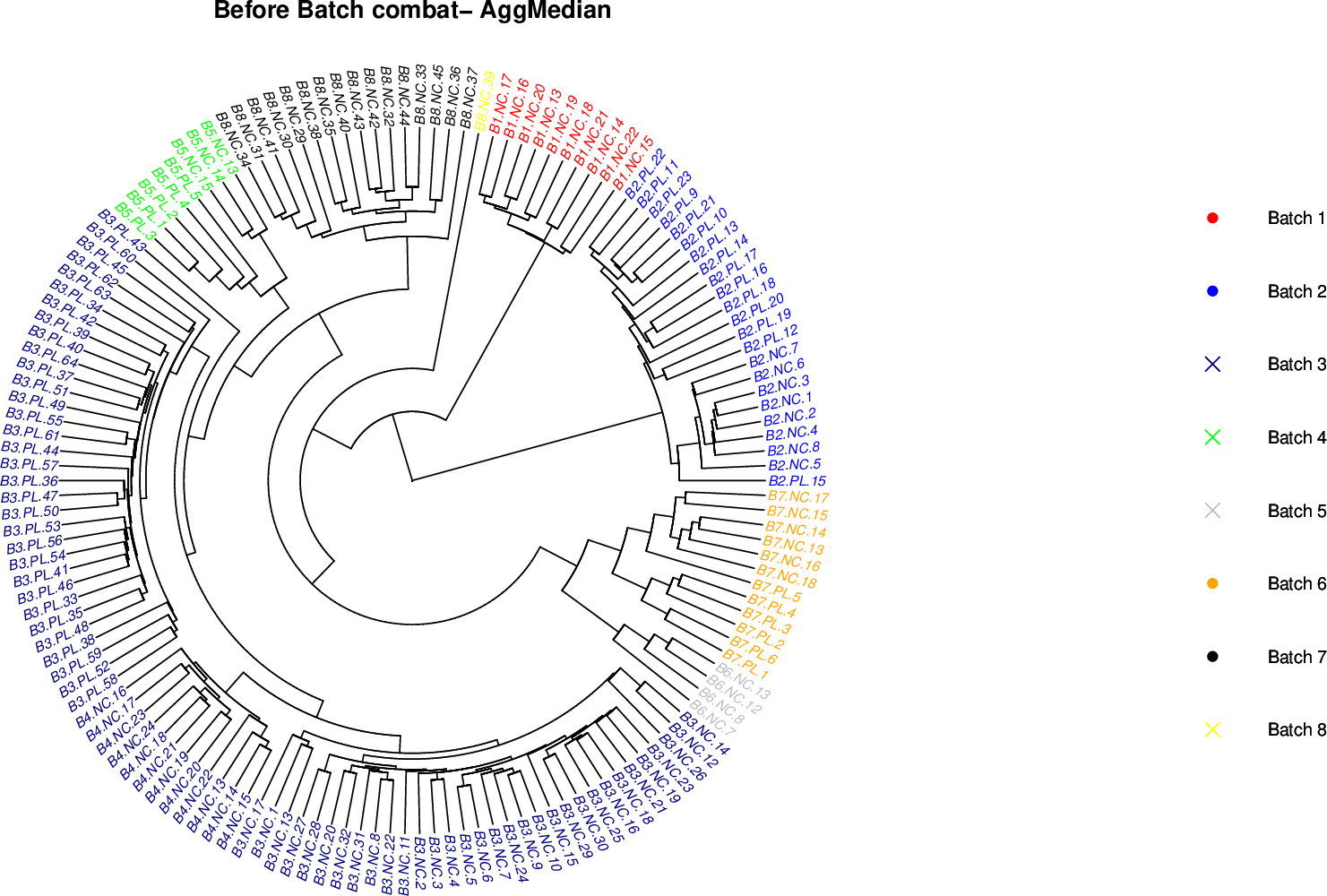

Supplement: S1 Fig — (TIF) [file pone.0267973.s001.tif]

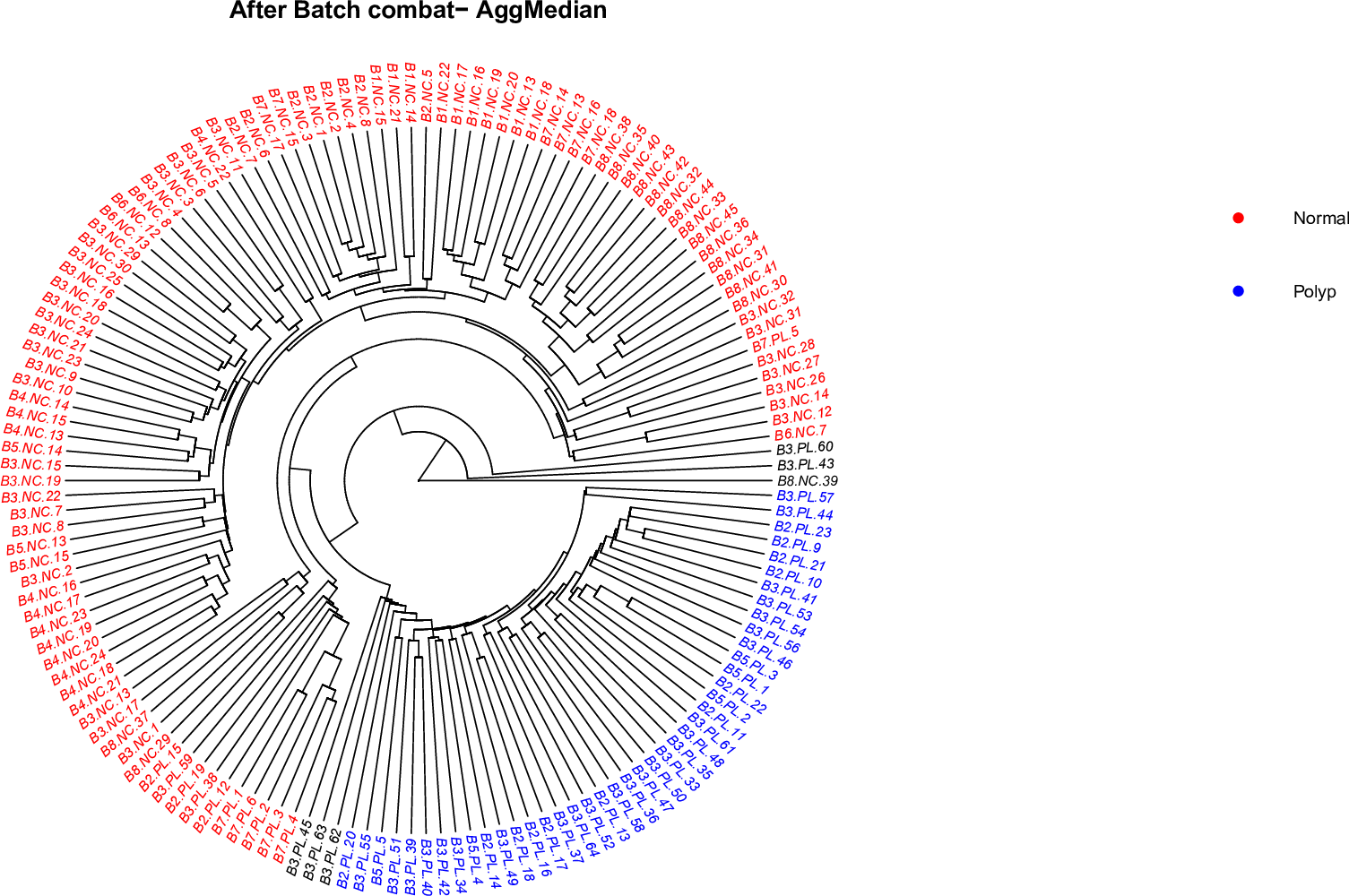

Supplement: S2 Fig — (TIF) [file pone.0267973.s002.tif]
