# Supplementary material for: In silico identification of novel biomarkers for key players in transition from normal colon tissue to adenomatous polyps
Source: PLoS One. 2022 Apr 29;17(4):e0267973. doi: 10.1371/journal.pone.0267973 (PMC9053805; doi:10.1371/journal.pone.0267973)
Supplement: S1 Table — (DOCX) [file pone.0267973.s003.docx]

| **Clustering algorithm** | **Gene symbol** | **Fold-change in validation** | **Regulation direction in validation** | **Fold-change in training** | **Regulation direction in training** |
| --- | --- | --- | --- | --- | --- |
| **FN 3^rd^ module** | ADAMDEC1 | -3 | down | -3.67 | down |
|  | APOE | -1.49 | down | -1.18 | down |
|  | BMP5 | -1.41 | down | -1.08 | down |
|  | CCL19 | -1.46 | down | -2.26 | down |
|  | CCL21 | -1.21 | down | -1.54 | down |
|  | CCL5 | -1.19 | down | -1.71 | down |
|  | CHGA | -2.42 | down | -3.38 | down |
|  | CHGB | -2.47 | down | -2.01 | down |
|  | CITED2 | -1.12 | down | -1.02 | down |
|  | CXCL12 | -1.58 | down | -1.99 | down |
|  | CXCL13 | -2.24 | down | -3.12 | down |
|  | F13A1 | -2.02 | down | -1.93 | down |
|  | GREM2 | -1.38 | down | -1.34 | down |
|  | HAPLN1 | -1.85 | down | -1.79 | down |
|  | IGF1 | -1.13 | down | -1.35 | down |
|  | MSX1 | 1.15 | up | 1 | up |
|  | NDN | -1.54 | down | -2.02 | down |
|  | NKX2-3 | -1.3 | down | -1.72 | down |
|  | OGN | -1.21 | down | -1.4 | down |
|  | PTN | -1.63 | down | -1.29 | down |
|  | PYY | -3.28 | down | -3.3 | down |
|  | SCG2 | -2.03 | down | -1.86 | down |
|  | SLIT2 | -1.35 | down | -1.01 | down |
|  | SST | -3.95 | down | -4.23 | down |
|  | STMN2 | -1.91 | down | -1.7 | down |
|  | TNFRSF17 | -1.63 | down | -1.94 | down |
| **FN 9^th^ module** | CSF2RB | -0.95 | down | -1.07 | down |
|  | IL10RA | -0.90 | down | -1.01 | down |
| **Spectral 1^st^ module** | CD14 | -0.97 | down | -1.44 | down |
|  | GPM6B | -1.52 | down | -1.17 | down |
|  | GPNMB | -1.73 | down | -1.32 | down |
|  | HLA-DPA1 | -1.23 | down | -1.42 | down |
|  | JAM2 | -1.24 | down | -1.14 | down |
|  | MS4A1 | -1.17 | down | -1.42 | down |
|  | NDN | -1.54 | down | -2.02 | down |
|  | PLP1 | -2.62 | down | -1.82 | down |
|  | SETBP1 | -1.28 | down | -1.47 | down |
|  | STMN2 | -1.91 | down | -1.7 | down |
|  | TLR4 | 0.78 | up | 1.10 | up |
|  | VCAM1 | -1.27 | down | -1.58 | down |
|  | CSF2RB | -0.95 | down | -1.07 | down |
|  | IL10RA | -0.90 | down | -1.01 | down |
