# Supplementary material for: In silico identification of novel biomarkers for key players in transition from normal colon tissue to adenomatous polyps
Source: PLoS One. 2022 Apr 29;17(4):e0267973. doi: 10.1371/journal.pone.0267973 (PMC9053805; doi:10.1371/journal.pone.0267973)
Supplement: S2 Table — (DOCX) [file pone.0267973.s004.docx]

| **Clustering algorithm** | **Gene symbol** | **Fold-change in validation** | **Regulation direction in validation** | **Fold-change in training** | **Regulation direction in training** |
| --- | --- | --- | --- | --- | --- |
| **FN** | ADAMDEC1 | -2.29 | down | -3.67 | down |
|  | CCL19 | -1.22 | down | -2.26 | down |
|  | CCL21 | -1.27 | down | -1.54 | down |
|  | CHGA | -1.23 | down | -3.38 | down |
|  | CITED2 | -1.5 | down | -1.02 | down |
|  | CXCL12 | -1.98 | down | -1.99 | down |
|  | CXCL13 | -1.38 | down | -3.12 | down |
|  | F13A1 | -1.32 | down | -1.93 | down |
|  | FHL1 | -2.64 | down | -1.31 | down |
|  | GREM2 | -1.5 | down | -1.34 | down |
|  | NDN | -1.29 | down | -2.02 | down |
|  | OGN | -1.29 | down | -1.4 | down |
|  | PYY | -1.7 | down | -3.3 | down |
|  | SLIT2 | -1.05 | down | -1.01 | down |
|  | SST | -1.38 | down | -4.23 | down |
|  | STMN2 | -1.22 | down | -1.7 | down |
|  | VIP | -1.46 | down | -1.12 | down |
| **Spectral** | GPM6B | -1.33 | down | -1.17 | down |
|  | GPNMB | -1.93 | down | -1.32 | down |
|  | NDN | -1.29 | down | -2.02 | down |
|  | PLP1 | -1.45 | down | -1.82 | down |
|  | SETBP1 | -1.42 | down | -1.47 | down |
|  | STMN2 | -1.22 | down | -1.7 | down |
